# Mesoporous Nano-Sized BiFeVOx.y Phases for Removal of Organic Dyes from Wastewaters by Visible Light Photocatalytic Degradation

**DOI:** 10.3390/nano12081383

**Published:** 2022-04-18

**Authors:** Niyazi A. S. Al-Areqi, Muhamad Umair, Ahmed M. Senan, Ahlam Al-Alas, Afraah M. A. Alfaatesh, Saba Beg, Kashif-ur-Rehman Khan, Sameh A. Korma, Mohamed T. El-Saadony, Mohammed A. Alshehri, Ahmed Ezzat Ahmed, Ahmed M. Abbas, Riyad A. Alokab, Ilaria Cacciotti

**Affiliations:** 1 Department of Chemistry, Faculty of Applied Science, Taiz University, Taiz 6803, Yemen; ahlamalalas@gmail.com (A.A.-A.); afraahmohammed35@gmail.com (A.M.A.A.); riad.aloqob@ye.liu.edu.ib (R.A.A.); 2 Department of Food Science and Engineering, College of Chemistry and Environmental Engineering, Shenzhen University, Shenzhen 518060, China; 3 Glycomics and Glycan Bioengineering Research Center, School of Food Science and Technology, Nanjing Agricultural University, Nanjing 210095, China; ahmedmsenan@njau.edu.cn; 4 Department of Chemistry, Aligarh Muslim University, Aligarh 202002, India; profsababeg@gmail.com; 5 Department of Pharmaceutical Chemistry, Faculty of Pharmacy, The Islamia University of Bahawalpur, Bahawalpur 63100, Pakistan; kashifur.rahman@iub.edu.pk; 6 Department of Food Science, Faculty of Agriculture, Zagazig University, Zagazig 44519, Egypt; sameh.hosny@zu.edu.eg; 7 Department of Agricultural Microbiology, Faculty of Agriculture, Zagazig University, Zagazig 44511, Egypt; m_tlatelsadony@yahoo.com; 8 Department of Biology, College of Science, King Khalid University, Abha 61413, Saudi Arabia; aabdelrahman@kku.edu.sa (M.A.A.); ahmed.ezzat@vet.svu.edu.eg (A.E.A.); ahmed.abbas76@gmail.com (A.M.A.); 9 Department of Theriogenology, Faculty of Veterinary Medicine, South Valley University, Qena 83523, Egypt; 10 Department of Botany and Microbiology, Faculty of Science, South Valley University, Qena 83523, Egypt; 11 Department of Engineering, INSTM RU, University of Rome “Niccolò Cusano”, Via Don Carlo Gnocchi 3, 00166 Roma, Italy; ilaria.cacciotti@unicusano.it

**Keywords:** BiFeVOx, photocatalyst, mesopores, dye degradation, waste water, photodegradation, adsorption

## Abstract

With an increasing demand for industrial dyes in our daily lives, water conditions have become worse. Recently, the removal of such environmentally hazardous pollutants from wastewaters through photocatalytic degradation has been drawing increased attention. Three mesoporous nanophases of BiFeVOx.y as (Bi_2_Fe^III^V_1−y_O_5.5−y_) visible light photocatalysts were synthesized in this study using ethylene glycol-citrate sol-gel synthesis combined with microwave- assisted calcination. X-ray diffraction (XRD), differential thermal analysis (DTA), FTIR spectroscopy, X-ray photoelectron spectroscopy (XPS), scanning electron microscopy coupled with energy dispersive X-ray spectrometry (SEM-EDS), nitrogen adsorption-desorption isotherms, and UV-Vis diffuse reflectance spectrophotometry (UV-Vis/DRS) were used to characterize the BiFeVOx.y photocatalysts. The visible light-induced photocatalytic activities of the BiFeVOx.y phases were evaluated by the degradation of methylene blue (MB) dye in aqueous solution at pH ~10.0. The results of this study show that the combination of doping strategy with the utilization of advanced synthesis methods plays an important role in improving the structure and surface properties of BiFeVOx.y phases, and thereby enhancing their adsorption and photocatalytic efficiencies. The synthesized mesoporous tetragonal γ-BiFeVOx.y nanophase has been proven to be a potential visible-light photocatalyst for the degradation of organic dyes.

## 1. Introduction

Synthetic dyes comprise an important part of industrial water effluents as they are discharged in abundance by many manufacturing industries. The impact of these dyes on the environment is a major concern because of the potentially carcinogenic properties of these chemicals [1,2,3,4,5,6]. Many treatment methods have been recently developed and applied to remove these dyes from wastewater, such as adsorption, ultrafiltration, reverse osmosis, coagulation, and ion exchange [7,8,9,10,11,12,13]. However, such methods do not result in the destruction of the dye molecules [14,15,16,17]. Therefore, much attention has been paid to finding an alternative to conventional methods such as “advanced oxidation processes” (AOPs), which are based on the chemical, photochemical, and photocatalytic production of hydroxyl radicals (OH^•^), which act as strong oxidizing agents for the degradation of organic dyes [18]. The advantages of AOPs over competing processes are: complete mineralization; no waste disposal problem; low cost; and only mild temperature and pressure conditions [19,20,21,22]. Many metal oxides, such as TiO_2_, ZnO, ZrO_2_, and so forth, have been widely investigated in the AOPs for the photodegradation of synthetic dyes in wastewater [20,23,24,25]. However, because of their relatively high band-gap energy, they are activated only by ultraviolet light absorption [26,27], which limits their commercial application for water and wastewater treatment. To overcome this problem, the visible light-induced photocatalysts are required to apply the AOPs more efficiently and to degrade these dyes using viable sunlight energy.

Our previous research on the electrical properties of oxide–ion conductors, namely BiMeVOx.y (Bi = bismuth, Me = dopant metal ion, V = vanadium, Ox = oxide, and y = molar dopant concentration), derived by the partial substitution of Me for V in the parent compound, Bi_2_VO_5.5_, of a layered Aurivillius–type structure, discovered that many BiMeVOx materials behave as semiconductors at temperatures less than 300 °C [28,29,30,31,32]. The first attempt to employ such types of materials as visible light photocatalysts was made by Thakral and Uma [33] by investigating the visible light photocatalytic efficiencies of Bi_2_VO_5.5_ and its substituted analogues, i.e., BiAlVOx, and BiGaVOx, for the degradation of methylene blue (MB) in aqueous media. These were found to be moderate for MB photodegradation, and showed similar activities of Bi_2_AlVO_7_ and Bi_2_GaVO_7_ photocatalysts [17]. Another investigation was also carried out with the BiNiVOx analogue on the photodegradation of a synthetic azo dye [34,35,36], which showed a considerably high photocatalytic efficiency, even though it was synthesized via the conventional solid–state reaction. Moreover, a possible photocatalytic degradation mechanism was proposed, and clearly presented. However, Chen et.al. [37] studied the photocatalytic activities of hierarchical Bi_2_VO_5.5_ hollow microspheres synthesized by the solvothermal route for the photodegradation of rhodamine-B (RhB), finding them to be much higher compared to other Bi_2_VO_5.5_ powders prepared by conventional synthesis routes.

Although some doped Bi_2_VO_5.5_ materials have shown enhanced photocatalytic efficiencies for the degradation of some organic dyes within the visible light spectral range as a consequence of band gap narrowing, there is no clear, convincing evidence to realize an actual relationship between the phase stability, nanostructure and photocatalytic efficiency of BiMeVOx photocatalysts [38,39]. So, in this paper, we report another new member of the BiMeVOx.y family, viz., BiFeVOx.y, ${\mathrm{Bi}}_{2}{\mathrm{Fe}}_{y}^{\left(\mathrm{III}\right)}V_{1-y}O_{5.5-\left(3y/2\right)},$ used for the photocatalytic degradation of MB dye under visible light irradiation. This study was devoted to carefully investigating the influence of metal doping on the phase stability and photocatalytic properties of the BiFeVOx.y photocatalyst, to provide a more detailed investigation on the correlation of the enhanced photocatalytic efficiencies with the nanostructured BiFeVOx.y phases porosity.

Three mesoporous nanophases (α-monoclinic, β-orthorhombic, and γ-tetragonal) of BiFeVOx.y were prepared by means of ethylene glycol-citrate sol-gel synthesis followed by microwave-assisted calcination. In this investigation, sophisticated analytical methods, such as X-ray diffraction (XRD), differential thermal analysis (DTA), Fourier transform infrared spectroscopy (FTIR), X-ray photoelectron spectroscopic (XPS), scanning electron microscopy (SEM), energy dispersive X-ray spectrometry coupled with scanning electron microscopy (EDS-SEM), nitrogen adsorption equipment and UV-Vis diffuse reflectance spectra (UV-Vis/DRS), were used.

## 2. Materials and Methods

### 2.1. Experimental

#### Preparation of BiFeVOx.y Phases

Analytical grade Bi(NO_3_)_3_.5H_2_O, NH_4_VO_3_ and Fe(NO_3_)_3_.9H_2_O (Sigma Aldrich, St. Louis, MO, USA) were used as starting materials without any further purification. In the present study, three different phases (α-monoclinic, β-orthorhombic, and γ-tetragonal) of BiFeVOx.y were prepared at three different compositions, y = 0.03, y = 0.07, and y = 0.15, respectively. It is interesting to note that the three main crystallographic phases of BiFeVOx.y were found to be effectively stabilized with a high purity at these dopant concentrations. Stock solutions of the starting materials (0.1 M) were prepared by dissolving an accurately weighed amount of corresponding material in deionized water. A 0.2 M citric acid (Sigma Aldrich, St. Louis, MO, USA) solution used as chelating agent was prepared in a deionized water–ethylene glycol mixture at a volumetric ratio of 3:1. A 0.5 M NH_3_ (Sigma Aldrich, St. Louis, MO, USA) solution was also used for adjusting the pH. The starting material’s solutions were mixed at a volumetric ratio of 2:y:(1 − y) = Bi:Fe:V with citric acid solution to form sol solutions. The ratio of citric solution to total metal ions was set at 1.5:1.0. The resulting sol solutions’ pH values were adjusted to ~7 by adding ammonia solution. The sol solutions were then heated at 80 °C under constant stirring for two hours to form visible gel. The conditions (Temperature and time) were established according to our previous work reported for another member of the BiMeVOx family that was synthesized by the same sol-gel route [40]. Wet gels were further dehydrated in an oven at 90 °C for 12 h to remove the excess water and obtain dried xerogels. The xerogel was thoroughly mixed in an agate mortar for further homogenization and was then subjected to a microwave-assisted calcination for 30 min in a modified microwave oven operated at a frequency of 2.45 GHz.

### 2.2. Characterization of Photocatalyst Samples

The phase structure of as-prepared BiFeVOx.y photocatalysts was investigated by XRD using a Rigaku/Max-B X-ray diffractometer (Rigaku Corporation, Tokyo, Japan) with Ni-filtered CuKα radiation (λ = 1.54060 Å). Data were recorded with the Bragg–Brentano geometry at a scan time of 0.6 s/increment in the range 5° ≤ 2θ ≤ 90°. The unit cell parameters were calculated by the Rietveld refinement method using the X’ Pert Plus software program. The average crystallite size was calculated from XRD line broadening via the Scherrer equation: D = 0.89λ/B cosθ, where D is the crystal size in nm, λ is the CuKα radiation wavelength (λ = 1.54060 Å), B is the half-width of the peak in radians and θ is the corresponding diffraction angle.

The DTA measurements were carried out with a Schimatzu SC-TA 60 thermal analyzer. A 20 mg- powdered sample of BiFeVOx.y was heated from 40 °C to 1000 °C at a constant heating rate of 10 °C min^–1^. The experiments were run in a nitrogen atmosphere supplied at a flow rate of 100 mL min^–1^.

The FTIR spectra of the synthesized compositions were collected on a Perkin Elmer spectrophotometer (PerkinElmer^®^, Waltham, MA, USA) in the transmittance mode over the wavenumber range of 4000–400 cm^–1^. BiFeVOx.y powders were diluted equally well with analytical grade KBr to 1.5 *w*/*w*%.

The XPS analysis of BiFeVOx.y phases was performed on a VG-ADES 400 instrument with MgKα (*hν* = 1253.6 eV) as the excitation source. A powdered sample of BiFeVOx.y was pre-treated in an O_2_ flow of 30 mL min^–1^ for 1 h at 500 °C, and was then outgassed for 30 min before being analyzed. The C 1s signal at 284.6 eV was taken as a reference for the binding energy calibration.

The morphologies and microstructures of the as-prepared photocatalysts were examined by scanning electron microscopy using a JEOL-6510 LV SEM (JEOL Ltd., Tokyo, Japan); the ImageJ program was employed for processing the reacquired SEM micrographs and calculating the average particle sizes. From energy dispersive X-ray spectrometry (EDS) coupled with the SEM instrument, the corresponding EDS profiles scanned over a full scale up to 20 kV were also taken for investigating the elemental compositions of the samples’ surfaces and checking their purity.

The nitrogen adsorption–desorption isotherms were collected in the nitrogen partial pressure range of 0.01 ≤ (*P/P_o_*) ≤ 0.99, by means of an Autosorb-1 (Quantachrome, Boynton Beach, FL, USA) adsorption at 77 K. The specific surface areas were calculated using the Brunauer–Emmett–Teller (BET) method, while the BJH model was applied for the pore size distribution.

The optical band-gap energy [41] of the as-prepared photocatalyst samples was estimated using the UV-Vis/DRS (Shimadzu, Tokyo, Japan). Spectral data were collected on a Shimadzu Scan UV-Vis spectrophotometer (UV-2450) at room temperature in the wavelength range 200–800 nm using BaSO_4_ as a standard reflectance reference. The direct band-gap energy values were determined by extrapolating the straight portion of (*αhν*)^2^ vs. (*hν*) plots to *α* = 0 point, by means of line-regression fitting to the Kubelka–Munk Equation (1) near the band-gap edge of absorption:

(1) $${\left(\alpha hv\right)}^{2}=A\left(hv-E_{g}\right)$$

where *α*, *h*, *ν* and *A* are the absorption coefficient, Planck constant, light frequency and absorption constant, respectively.

### 2.3. Dark Adsorption Measurements

Continuous adsorption equilibrium experiments were carried out to investigate the decolorization of MB solution by BiFeVOx.y photocatalysts in the dark. A 200 mg BiFeVOx.y sample was transferred into a 500 mL, 5.0 × 10^−5^ M MB solution at pH ~10.0 (adjusted with diluted aqueous solutions of NH_3_ and HCl). The resulting solution was magnetically stirred in the dark at a moderate speed and then allowed to sediment. At various time intervals, a 5 mL aliquot of the dye-photocatalyst suspension was centrifuged and the remaining dye concentration was determined using a Shimadzu UV-Vis spectrophotometer (UV-2450) at a maximum absorption wavelength (λ_max_ = 665 nm). The amount of MB adsorbed per unit mass of the photocatalyst at any time (*q_t_* (mg g^−1^)) was calculated using Equation (2):

(2) $$q_{t}=\frac{V\left(C_{o}-C_{t}\right)MW}{m}$$

where *C_o_* and *C_t_* are the initial and remaining concentrations of the MB solution, *MW* the molecular mass of MB (319.85 g mol^−1^; C_16_H_18_ClN_3_S), *m* the mass expressed in grams of the BiFeVOx.y photocatalyst added into a definite volume, and *V* the volume of the dye solution. The amount of MB adsorbed per unit mass of the photocatalyst at equilibrium (q_max_) and the equilibrium concentration of MB (*C_e_*) are equal to *qt*, and *C_t_*, respectively, at the adsorption equilibrium time.

### 2.4. Photocatalytic Degradation Measurements

The same amount of the BiFeVOx.y photocatalyst and of the volume of virgin MB solution at the same conditions were transferred into a 750 mL photoreactor, equipped with water refrigeration, magnetic stirrer, and air mini pump. The resulting suspension was then magnetically stirred in the dark for 25 min to reach the adsorption–desorption equilibrium. A 300-W xenon lamp, located beyond an optical glass cut–off filter, was used as the visible light source with wavelengths greater than 400 nm. The irradiation source was located 25 cm above the surface of liquid in the photoreactor. The temperature of the reaction system was kept at 25 °C using flowing cool water in order to prevent the thermal catalytic reaction effect. At equal time intervals of irradiation (10 min), 5 mL aliquot of the reaction mixture was withdrawn from the photoreactor and then centrifuged. The dye concentration versus irradiation time was determined by measuring the maximum absorbance at λ_max_ = 665 nm using a Shimadzu UV-Vis spectrophotometer (UV-2450). The photocatalytic degradation percentage (*PD%*) of MB after 120 min of irradiation was calculated using the relation (3):

(3) $$PD\%=\left(1-\frac{C_{t}^{\prime }}{C_{e}^{}}\right)$$

where *C_e_* and *C′_t_* are the equilibrium concentration and the concentration at irradiation time (t) of the MB solution, respectively. The photocatalytic activity of the BiFeVOx.y phases for the MB photodegradation was also investigated using the pseudo first–order kinetic model (4):

(4) $$PD\%=ln\left(\frac{C_{e}}{C_{t}^{\prime }}\right)=k_{app},$$

where *k_app_* denotes the apparent first–order rate constant.

## 3. Result and Discussion

**Figure 1:**
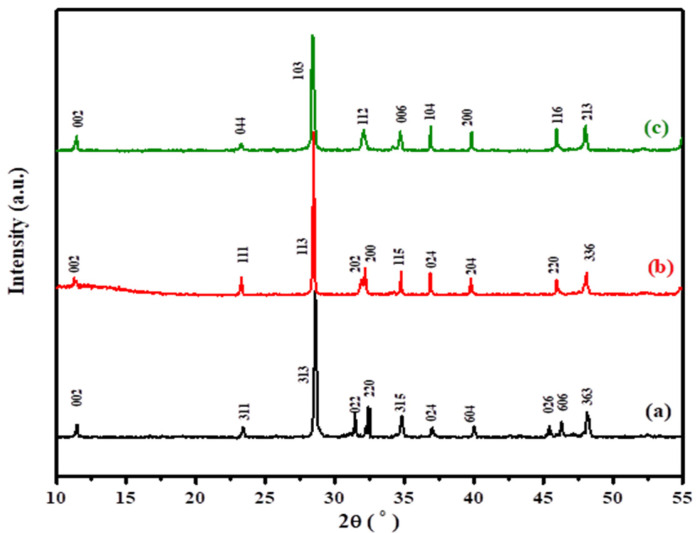
XRD patterns of the as–prepared BiFeVOx.y systems for (**a**) y = 0.03, (**b**) y = 0.07, and (**c**) y = 0.15.

Figure 1 presents the XRD patterns of the as-prepared BiFeVOx.y systems, identifying three principal crystallographic phases: the α-(monoclinic), β-(orthorhombic) and γ-(tetragonal) BiFeVOx.15 phases. The α-and β-phases with space group Aba2 and Acam are assigned to BiFeVOx.03 and BiFeVOx.07, respectively.

The singlet (112) sublattice peak at 2θ~32.5° is clear evidence for the room temperature stabilization of the tetragonal γ-BiFeVOx.15 phase. The values of refined unit cell parameters, crystallite size (D) and crystallographic density (dXRD) are summarized in Table 1. It can be observed that the lattice dimensions, particularly along the c-axis, remarkably increase with the Fe content (y). This is in fact attributed to the substitution of the smaller V^5+^ (0.54 Å) with the larger Fe^3+^ ion (0.78 Å) in the 6-coordination environment, adapted by the perovskite vanadate layers [42].

**Figure 2:**
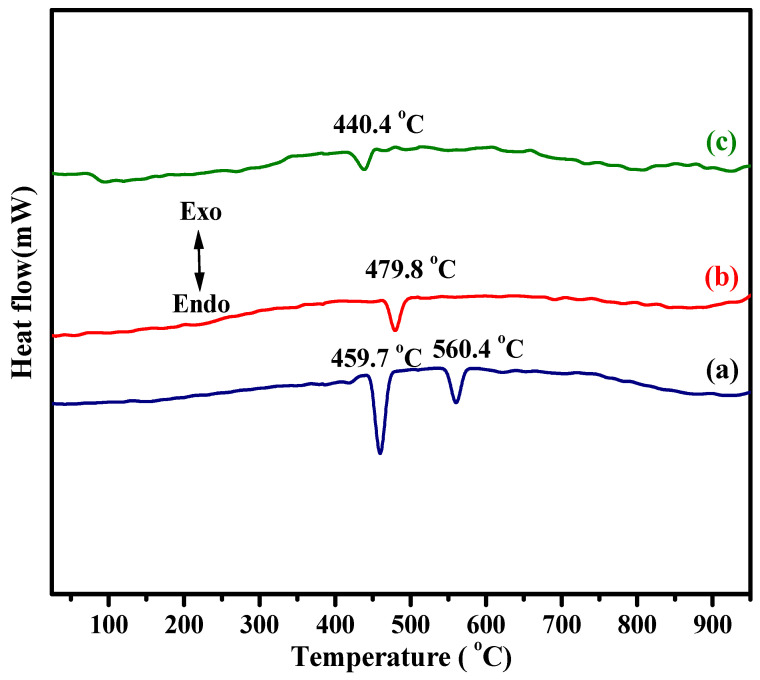
DTA thermograms of BiFeVOx.y for (**a**) y = 0.03, (**b**) y = 0.07, and (**c**) y = 0.15.

The endothermic peaks shown in the DTA thermograms of the BiFeVOx.y system (Figure 2) are in good agreement with the XRD assignment. Like α-Bi_2_VO_5.5_ [43] and α-BiMeVOxes [44,45], α-BiFeVOx.03 exhibited two typical endothermic peaks at 459.7 and 560.4 °C due to the successive α →β→γ′ (high– temperature fully disordered tetragonal) transitions, while the single peaks observed for BiFeVOx.07 and BiFeVOx.15 can be ascribed to β→γ′ and γ→γ′, respectively.

**Figure 3:**
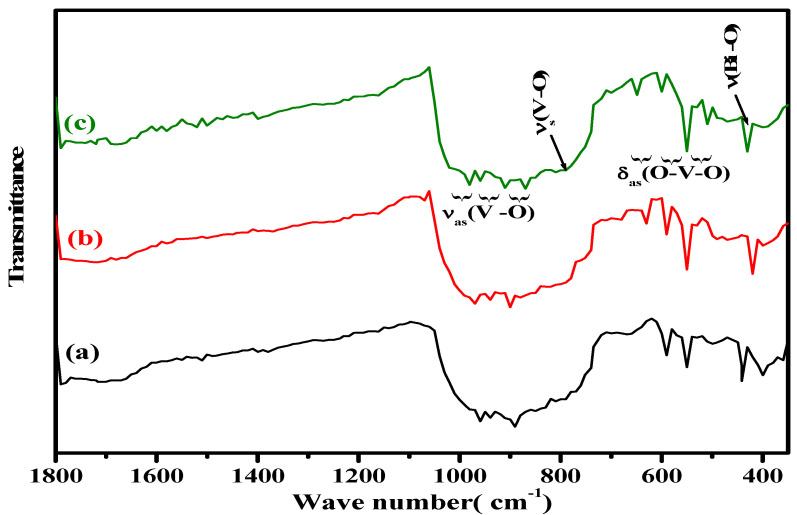
FTIR spectra of BiFeVOx.y for (**a**) y = 0.03, (**b**) y = 0.07, and (**c**) y = 0.15.

FTIR spectra of the as–prepared BiFeVOx.y compositions (Figure 3) show absorption bands in the regions 700–720, 800–990, and 510–620 cm^–1^ which are assigned to symmetric ν_s_(V-O), and asymmetric ν_as_(V-O) stretching modes of vibration, and asymmetric deformation modes of bending δ_as_(O-V-O) for the vanadate layers, respectively. Moreover, another band can be observed at ~460 cm^–1^, ascribed to Bi/Fe-O stretching vibrations. Similar typical FT-IR assignments have also been reported for other BiMeVOxes prepared via the conventional solid synthesis [46].

**Figure 4:**
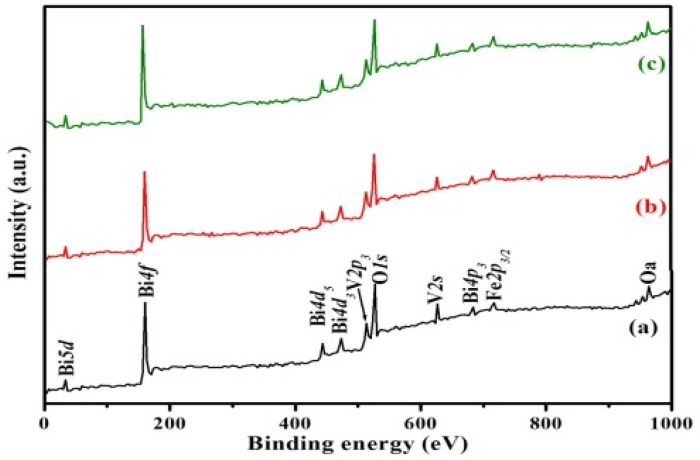
XPS profiles of BiFeVOx.y for (**a**) y = 0.03, (**b**) y = 0.07, and (**c**) y = 0.15.

Figure 4 illustrates XPS profiles of the three BiFeVOx.y phases. All peaks shown here are assigned to Bi, V, Fe, and O atoms as the main surface species of as-prepared BiFeVOx.y compositions with single oxidation states of +3, +5, +3, and −2, respectively. XPS analysis confirms that the BiFeVOx.y compositions are of high purity and free of any carbon contamination and, thus, exist in single phases as already revealed by XRD results. It is interesting to note that the slight shifts in XPS peaks corresponding to angular V2p and spin V2s momentum towards higher binding energies with increasing Fe content are mainly caused by the partial substitution of Fe for V in the perovskite vanadate layers.

However, the very small differences between the atomic percentages of O and V obtained from the XPS results (Table 2), and those theoretically deduced from the corresponding formulas are in good agreement with the fact that such materials are oxygen deficient and that the oxygen vacancies are entirely located in the vanadate layers [39,47], i.e., [V_1–y_Fe_y_O_3.5–y–δ_ ❑_δ_]$\begin{array}{c} 2n-\\ n \end{array}$*,* where ❑ and δ stand for an oxygen vacancy and its molar ratio. Moreover, the strong peak, with two little overlapped shoulders ascribed to the O1s spin momentum, indicates three different oxygen environments in the crystal structure. In detail, the strong peak is associated with the bismuthate layers ([Bi_2_O_2_]$\begin{array}{c} 2n+\\ n \end{array}$), whereas the two shoulders correspond to apical and equatorial oxygen atoms in the perovskite vanadate layers, having two principal types of Fe/V polyhedral, i.e., distorted tetrahedra and distorted octahedra. This is in a good agreement with the previous detailed studies reported for the crystal defect structure determinations of BiMeVOx.y [48,49]. 

**Figure 5:**
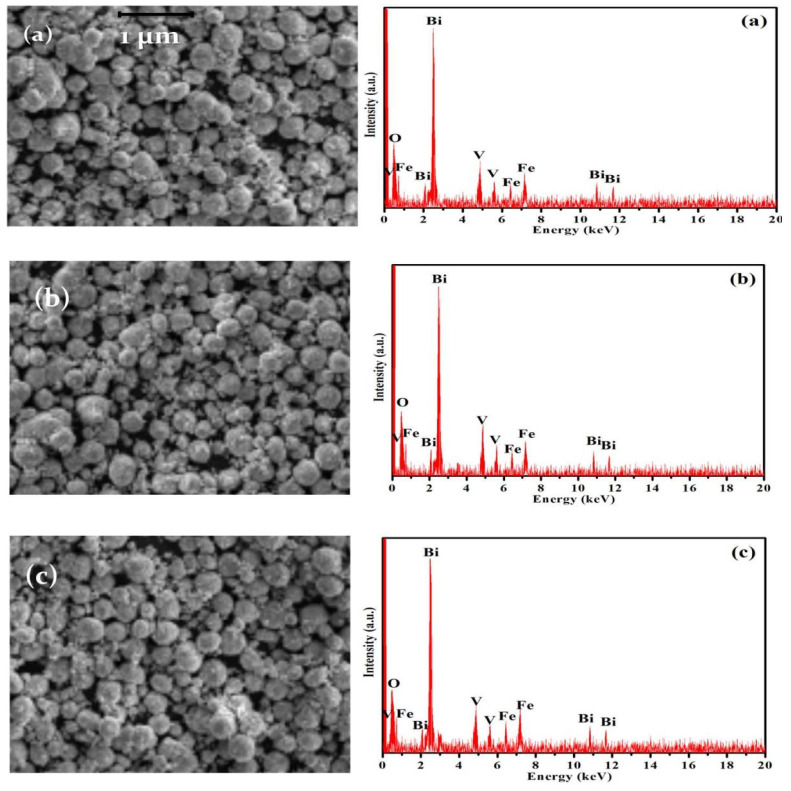
SEM micrographs (**left**) and EDS profiles (**right**) of BiFeVOx.y for (**a**) y = 0.03, (**b**) y = 0.07, and (**c**) y = 0.15.

SEM micrographs and the related EDS profiles of as–synthesized BiFeVOx.y phases are presented in Figure 5. It can be observed that the major particles of all samples exhibit a spherical-like shape, while the minor very tiny particles of irregular shapes are spread out at the boundaries of the spherical particles. The size distribution of spherical particles ($\bar{d}$) was measured to be several hundreds of nanometers (Table 2). Interestingly, the values of the Fe/V molar ratio estimated by the EDS analysis well match with those obtained from the XPS results.

**Figure 6:**
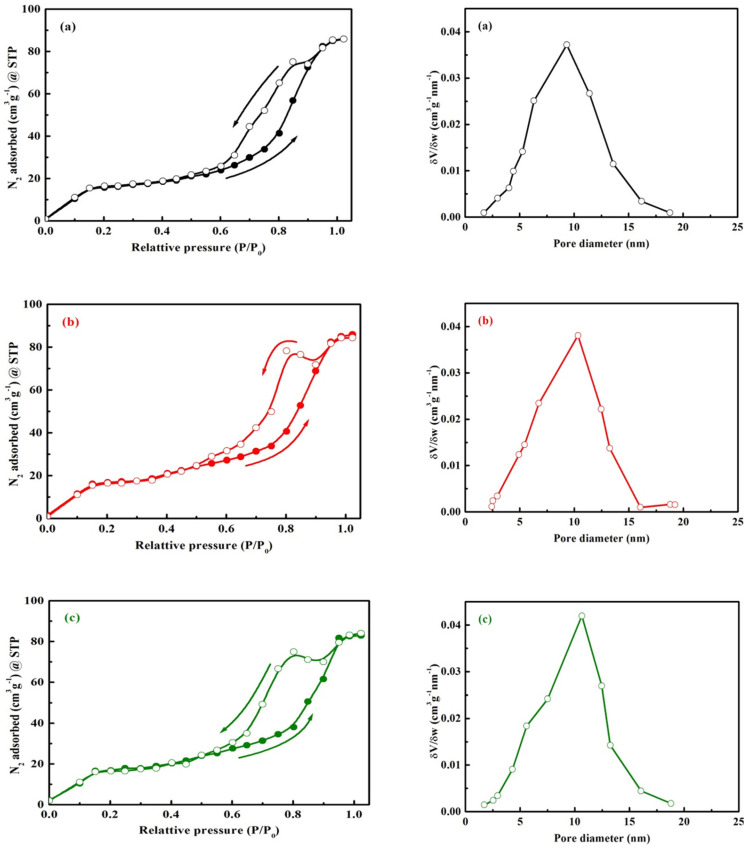
N_2_ adsorption-desorption isotherms (**left**) and pore size distribution curves (**right**) of BiFeVOx.y for (**a**) y = 0.03, (**b**) y = 0.07, and (**c**) y = 0.15.

N_2_ adsorption–desorption isotherms and BJH pore size distribution curves of BiFeVOx.y phases are shown in Figure 6. According to the IUPAC classification, the adsorption isotherms are all of type IV with a remarkable hysteresis loop upon the desorption run. This is a typical feature of powders containing disordered mesopores finely intra–aggregated within the major spherical particles [50,51]. It is also observed that all three phases exhibit a narrow pore size distribution with a characteristic maximum.

The estimated values of BET specific surface area (S_BET_), pore volume, and average pore diameter are summarized in Table 3. It is worthwhile to appoint here that the specific surface area of the mesoporous BiFeVOx.y system is nearly 160 times greater than that estimated for the polycrystalline BiNiVOx system synthesized by the solid state reaction [34]. The S_BET_, pore volume and pore diameter of BiFeVOx.y systems were found to be remarkably increased with e content. The significant increment of these microstructural properties could be attributed to an enhanced coalescence process, since the microwave-assisted calcination is favored by the presence of Fe dopant, which is a good microwave absorber.

**Figure 7:**
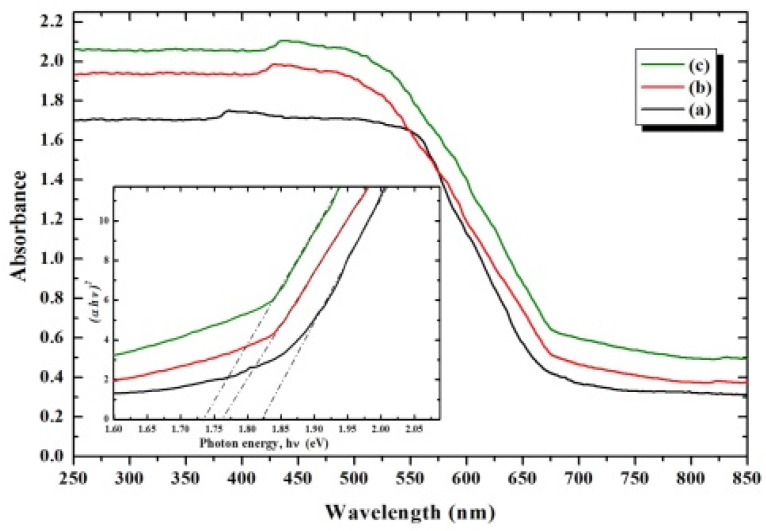
UV-Vis/DR spectra of BiFeVOx.y for (**a**) y = 0.03, (**b**) y = 0.07, and (**c**) y = 0.15. Inset shows (*αhυ*)^2^ vs. *hυ* plots.

Figure 7 shows the UV-Vis/DR spectra of as–prepared BiFeVOx.y phases. The characteristic absorption edge shifts toward higher wavelengths as the Fe content increases, due to the lowering in the electronic transition energy between the valence band (VB) and conduction band (CB). This is reflected by the variation of optical band-gap energy [14] with the composition (Table 4) as estimated from (*αhν*)^2^ vs. *hν* plots, depicted as the inset of Figure 7.

**Figure 8:**
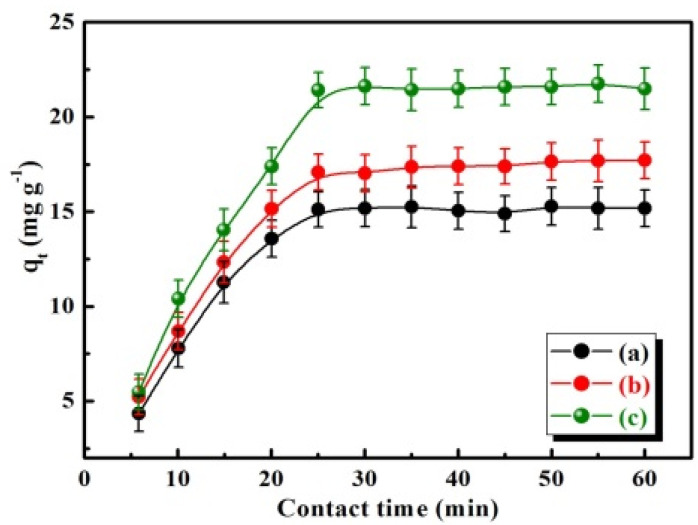
Time- dependence of MB adsorption onto BiFeVOx.y for (**a**) y = 0.03, (**b**) y = 0.07, and (**c**) y = 0.15, in the dark.

Prior to performing the photocatalytic degradation experiments, it was necessary to determine the equilibrium time and bulk dye concentration for the MB adsorption onto BiFeVOx.y samples in the dark. Figure 8 illustrates the amount of MB adsorbed (*q_t_*) vs. contact time [52] plots for the MB adsorption onto BiFeVOx.y samples (500 mL, 5.0 × 10^–5^ M + 200 mg BiFeVOx.y), in the dark.

It is observed that 25 min was sufficient to reach the adsorption equilibrium. The estimated values of equilibrium time [14], bulk MB concentration [15] and the maximum amount of MB adsorbed at equilibrium (q_max_) are listed in Table 3 as the mean ± SD derived from three replications. The variations of *C_e_* and q_max_ with Fe content well agree with the results obtained from N_2_ desorption isotherms.

**Figure 9:**
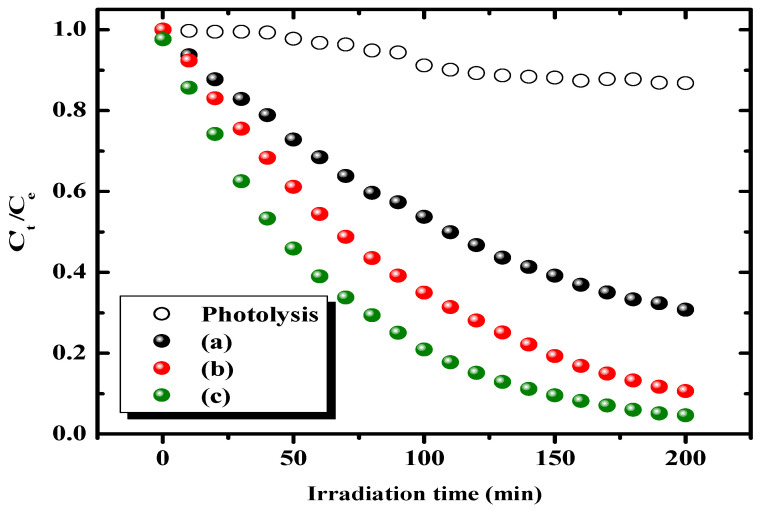
Photocatalytic MB degradation in aqueous medium using BiFeVOx.y catalysts for (**a**) y = 0.03, (**b**) y = 0.07, and (**c**), y = 0.15, under visible light irradiation.

The variations of photocatalytic activity of the BiFeVOx.y catalyst series for MB degradation under visible light irradiation in comparison with MB photolysis are illustrated in Figure 9. The values of photocataltyic degradation percent (*PD%*) after 2 h of irradiation are listed in Table 4.

**Figure 10:**
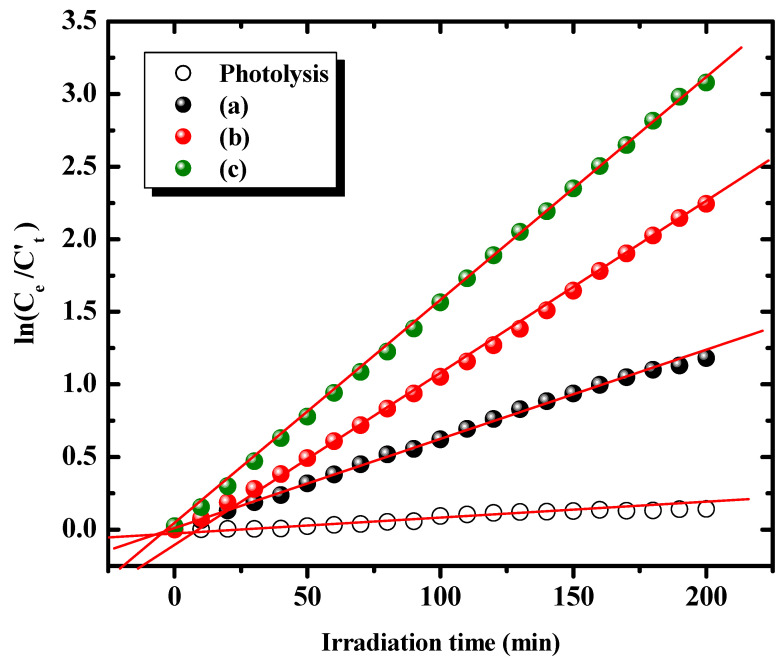
Pseudo first-order kinetics of MB photodegradation catalyzed by BiFeVOx.y for (**a**) y = 0.03, (**b**) y = 0.07, and (**c**) y = 0.15, under visible light irradiation.

It is observed that the *PD*% remarkably increases with Fe dopant content, reaching as much as 84.86% in the presence of the γ-BiFeVOx.15 phase. The line-regression fitting of *ln* (*C_t_*/*C_e_*) vs. irradiation time plots for visible light MB photodegradation catalyzed by the BiFeVOx.y system can be seen in Figure 10.

The estimated *k_app_* values (Table 4) indicate that the photocatalytic MB degradation proceeds more rapidly with β-BiFeVOx.07 and γ-BiFeVOx.15 compared with the α-phase, which is consistent with the variation of band-gap energies and surface properties of investigated BiFeVOx.y phases.

**Figure 11:**
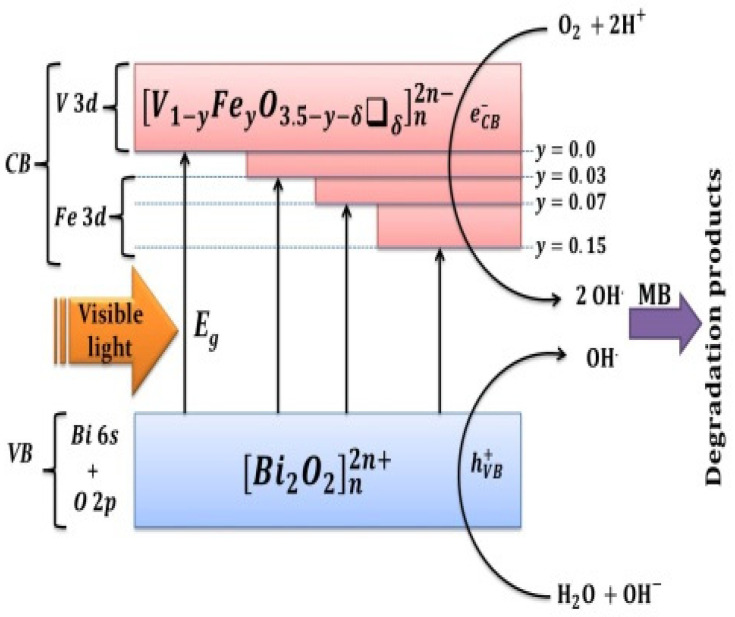
Schematic representation of photocatalytic degradation reactions of MB upon visible- light irradiation of the BiFeVOx.y system.

The interesting point to be emphasized here is that Bi6s and O2p orbitals of the bismuthate layers constitute the electronic structure of VB, while that of CB is dominantly composed of V3d and Fe3d orbitals of the perovskite vanadate layers [33,34,53]. Accordingly, the reduction in the band-gap energy of the BiFeVOx.y system with increasing Fe doping is mainly attributed to the additional contribution of Fe_3_d orbitals in the CB bottom lowering (Figure 11).

This reasonably supports our proposed mechanism for the visible light photocatalytic efficiency of BiMeVOx materials [34], suggesting the crucial role of highly disordered oxygen vacancies of the perovskite vanadate layers in generating an electron trap under the CB to narrow band gaps. Thus, this occurrence restrains the recombination of photogenerated holes and electrons and consequently improves the visible light photocatalytic activity. Figure 11 presents a summary of the MB degradation photocatalytic reactions upon visible-light irradiation of the BiFeVOx.y system. It is also interesting to note that, in addition to their electronic contribution to the photocatalytic activity, such oxygen vacancies participate, to a great extent, in increasing surface defects/active sites available for the adsorption of more dye molecules, via the synthesis of mesoporous particles [37,54,55,56]. Therefore, the use of the ethylene glycol–citrate sol–gel method coupled with microwave-assisted calcination in the present study has shown significantly improved photocatalytic efficiencies of synthesized mesoporous nano BiFeVOx.y phases.

## 4. Conclusions

In this work, three BiFeVOx.y mesoporous nanophases were successfully synthesized and tested as visible light photocatalysts. We demonstrated a facile approach for the followed synthesis procedure by ethylene glycol-citrate sol-gel synthesis coupled with microwave- assisted calcination. The highly pronounced photocatalytic efficiency of mesoporous tetragonal γ-BiFeVOx._15_ nanophase indicates that the photocatalytic performance of BiMeVOxes with a layered perovskite structure can be further improved by the doping strategy. Therefore, the combination of doping strategy with such a facile synthesis method played an important role in improving the structure and surface properties of BiFeVOx.y phases and, thereby, enhancing their adsorption and photocatalytic efficiencies. Therefore, the present work proposes a rationalized approach to fabricating and designing potential visible light BiMeVOx-based photocatalysts. However, the photocatalytic degradation mechanism should be proposed and considered in the near future after carrying out advanced trapping experiments of degradation intermediates and final products, along with the reuse of BiFeVOx.y for the photocatalysis.

## Figures and Tables

**Table 1:** Stabilized phase, refined unit cell parameters, crystallite characteristics of as–prepared BiFeVOx.y photocatalyst series.

*y*	Stabilized Phase		Unit Cell Parameters			Crystallite Parameters	
	Phase	Space Group	*a* (Å)	*b* (Å)	*c* (Å)	*D* (μm)	d_XRD_ (g cm^3^)
0.03	α	*Aba2*	5.538	5.596	15.338	1.93	6.56
0.07	β	*Acam*	5.614	5.514	15.362	2.04	6.52
0.15	γ′	*I4/m mm*	3.971	-	15.416	1.87	6.48

**Table 2:** Chemical analysis and microstructure characteristics of the as–prepared BiFeVOx.y photocatalyst series.

*y*	XPS (Atomic%)				SEM	
	Bi%	V%	Fe%	O%	$\bar{d}$(nm)	Fe/V Mole Ratio
0.03	23.61	11.46	0.35	64.57	337.65	0.0302
0.07	23.72	11.05	0.83	64.39	308.91	0.0747
0.15	23.95	10.20	1.80	64.05	306.32	0.1762

**Table 3:** Specific surface characteristics and dye adsorption efficiencies of BiFeVOx.y photocatalyst series.

*y*	N_2_ Adsorption-Desorption				MB Adsorption				
	S_BET_ (m^2^ g^−1^)	R^2^	Pore Diameter (nm)	Pore Vol. (cm^3^ g^−1^)	t_e_ (min)	q_max_ (mg g^−1^)	SD	*C_e_* (×10^−5^ M)	SD (×10^−7^)
0.03	58.813	0.9948	9.292	0.347	25	15.17	±0.94	3.11	±2.41
0.07	60.348	0.9965	10.345	0.395	25	17.06	±1.12	2.62	±1.83
0.15	65.671	0.9937	10.642	0.448	25	21.52	±1.43	2.31	±1.16

**Table 4:** Optical band-gap energies and photocatalytic efficiencies of BiFeVOx.y photocatalyst series.

*y*	UV-Vis/DR			MB Photodegradation		
	*E_g_* [22]	R^2^	SD	*k_app_* (min^−1^)	R^2^	*PD%*
0.00	2.19	0.9983	±0.019	8.58 × 10^−4^	0.9896	10.74
0.03	1.82	0.9966	±0.028	6.05 × 10^−3^	0.9987	53.28
0.07	1.76	0.9991	±0.017	0.01142	0.9992	71.92
0.15	1.73	0.9989	±0.021	0.01559	0.9998	84.86

## Data Availability

The data presented in this study are available on request from the corresponding author.
